# Nettle Leaf Water Extracts for Hepatoprotection: Insights into Bioactivity and Mitochondrial Function

**DOI:** 10.3390/plants14070992

**Published:** 2025-03-21

**Authors:** Ruta Muceniece, Beatrise Luize Revina, Jorens Kviesis, Aris Jansons, Kirills Kopiks, Kaspars Jekabsons, Kristine Saleniece, Jana Namniece, Zane Grigale-Sorocina, Baiba Jansone

**Affiliations:** 1 Faculty of Medicine and Life Sciences, University of Latvia, LV-1004 Riga, Latvia; 2 Faculty of Science and Technology, University of Latvia, LV-1004 Riga, Latvia; jorens.kviesis@lu.lv; 3 Kinetics Nail Systems, Ltd., LV-1067 Riga, Latvia

**Keywords:** nettle leaf extracts, hepatoprotection, phytochemicals, antioxidants, mitochondria, oxygraphy, oxidative stress, AGE inhibition

## Abstract

This study aimed to evaluate the hepatoprotective effects of nettle (*Urtica dioica* L.) leaf water extracts on oxygen consumption in the fatty acid oxidation (FAO) pathway using an in vitro fatty liver HepG2 cell model and employing an oxygraphy approach. It also examined the impact of these extracts on HepG2 cell lipid accumulation and viability under oxidative stress. The extracts were obtained via maceration with preservatives or by sonication with/without preservatives. Their chemical composition, including polyphenols, vitamins, and minerals, was analyzed. Bioactivity was confirmed through antioxidant and antiglycation in vitro assays. The extracts contained minerals, water-soluble vitamins, and polyphenols, primarily phenolic acids and rutin. Sonication increased the polyphenol yield, advanced glycation end-product (AGE) inhibition, and total antioxidant capacity compared to maceration. The added preservatives enhanced DPPH scavenging, while SOD-mimicking effects were comparable across extraction methods. In the liver steatosis model, the nettle extracts improved HepG2 cell viability under oxidative stress, reduced lipid accumulation, and enhanced mitochondrial oxygen consumption in the FAO pathway at mitochondria complex I. These findings demonstrate the impact of nettle leaf water extracts on oxygen flux in different oxidative phosphorylation states of the FAO pathway and deepen the understanding of nettle’s protective role in hepatic steatosis. The obtained results confirm the hepatoprotective effects of nettles through multiple mechanisms, primarily involving antioxidant activity, modulation of lipid accumulation, and mitochondrial protection.

## 1. Introduction

Stinging nettles (*Urtica dioica* L.) are widespread throughout Europe, North America, North Africa, and some parts of Asia [1,2,3]. This plant has a rich history as a wild vegetable with a high nutritional value and as a natural remedy. In general, nettle leaf and root extracts have a demonstrable wide range of effects, including hypoglycemic, hypolipemic, analgesic, antibacterial, antiviral, anti-Alzheimer’s, anticarcinogenic, anti-colitis, anti-inflammatory, antioxidant, anti-proliferative, and anti-rheumatic effects [1,2,3,4,5]. Comprehensive reviews have highlighted the positive effects of nettle consumption on metabolic conditions, namely, an increase in lipid metabolism and fatty acid oxidation (FAO), regulation of lipid peroxidation, and a decrease in oxidative stress in the liver [6,7]. Excessive absorption of lipids and fatty acids leads to the formation and storage of lipid droplets in liver cells. Accumulation of these droplets causes non-alcoholic liver disease (NAFLD), which can progress to pathological stages, including fibrosis and cancer. Fatty acid-induced lipid droplets are the first sign of steatosis, followed by oxidative stress and inflammation. These features of NAFLD are used in in vitro models by exposing primary hepatocytes or different liver cell lines to oleic acid and palmitic acid to induce lipid accumulation [8,9,10].

Nettles have a long history of traditional use and are of particular interest at present, with studies focusing on their medicinal benefits and uses in the cosmetic and food industries. As the pharmaceutical and cosmetic industries continue to advance, there is a growing research interest in potential pharmacological applications of nettle extracts, their biological effects, and the refinement of extraction methods. These research interests have led to the identification of key chemically active compounds present in various parts of the nettle (leaf, root, and flower), such as polyphenolic compounds, vitamins, essential amino acids, fatty acids, carotenes, and terpenoids, thereby enabling the targeted extraction and use of plant components to optimize nutritional and pharmacological activity [4,5,6,7,11].

Effectively extracting biologically active substances may require aggressive physical agitation and/or harsh solvents. Additionally, proper by-product disposal involves distilling used organic solvents, and the increased waste management demands can be a limiting factor for extract production by manufacturers. These considerations have driven advancements in extraction procedures, leading to the development of many innovative techniques, including microfiltration, pulsed electric field treatment, high-pressure processing, microwave-assisted extraction, enzymatic extraction, supercritical fluid extraction, supercritical CO_2_ extraction, and ultrasound-assisted extraction (sonication). Studies on ultrasound-assisted extraction’s key parameters, such as extraction time, amplitude, and the sample-to-solvent ratio, have allowed for the identification of optimal conditions for the recovery of bioactive compounds from pecan nutshell waste biomass, promoting sustainable development by minimizing environmental degradation and conserving natural resources [12]. Methanol and ethanol extracts from safflower by-products, including leaves, stems, and a leaf–stem mixture, obtained through sonication exhibited an antioxidant-rich chemical composition that inhibited erythrocyte hemolysis in vitro [13]. These breakthroughs collectively fall under the umbrella of “green recycling” and have a shared emphasis on simplicity, energy efficiency, and economic viability [11,12,13,14,15,16,17].

The advent of these innovative technologies and the escalation in consumer demand have resulted in a decline in the use of the time-consuming conventional maceration method of extraction. Ultrasonic methods enable the rapid preparation of extracts and expand the range of possible solvents to include water. Water extracts are favored as they offer a straightforward and cost-effective technique that eliminates organic solvents in polyphenol extraction [11,15,16,17]. For applications in beverage and food production, there is a significant preference for water extracts. Furthermore, some studies have indicated that aqueous nettle extracts exhibit a phenolic content over ten times higher than that of methanol, chloroform, diethyl ether, ethyl acetate, and butanol extracts [18]. For mint and nettle leaves, glycerol–water systems and elevated temperatures have been highlighted as alternatives to more commonly utilized water–ethanol extraction methods [19].

There is no one single ingredient responsible for nettles’ activities. Most of the research conducted thus far has focused on crude nettle leaf, root, and flower extracts. Chemical analysis of stinging nettles has shown the presence of different classes of chemical compounds, such as terpenes, minerals, vitamins, fatty acids, carotenes, polyphenolic compounds, amino acids, and many others that are considered beneficial for human health [20,21]. Furthermore, the antioxidant activity of nettle extracts is believed to support most biological activities [22]. Different analytical techniques, combined with spectrophotometric analysis and various sample preparation techniques, have been used to uncover the chemical composition of nettle extracts, with particular attention paid to the contents of polyphenols, phenolic acids, carotenoids, and minerals due to their significant biological activities [11,23].

**Figure 1:**
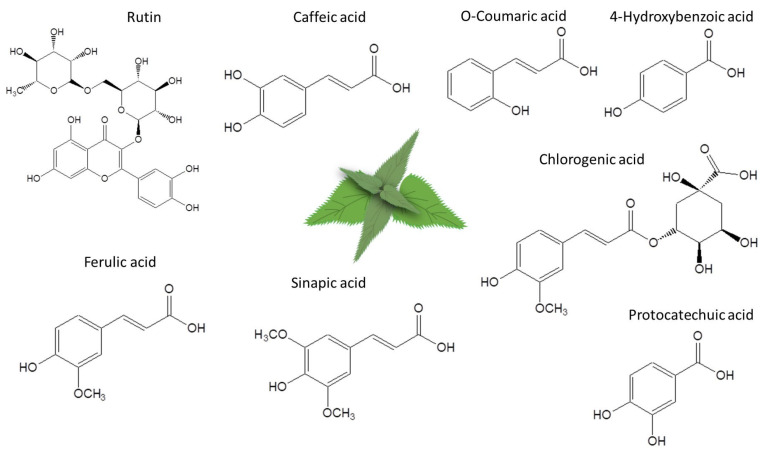
Main phenolic compounds obtained from the nettle leaf water extracts.

The primary objective of this study was to expand research on the hepatoprotective properties of nettle leaf water extracts using an in vitro HepG2 human cell line liver steatosis test model. Although nettle water extracts have been studied for their potential health benefits, including their antioxidant properties and effects on cellular metabolism, there is a lack of studies examining their impact on liver cell mitochondrial function in steatosis using an oxygraphy approach. Therefore, we incorporated high-resolution respirometry measurements of oxygen consumption in the fatty acid oxidation (FAO) pathway of HepG2 cell mitochondria into our study. To obtain nettle leaf water extracts, we used maceration or sonication techniques aimed to assess, quantify, and compare the chemical composition, as well as the antioxidant and antiglycation effects, of nettle leaf water extracts—with and without preservatives—to elucidate the bioactivity of the obtained extracts (Figure 1).

## 2. Results

### 2.1. Chemical Composition of the Nettle Leaf Water Extracts

As seen in Table 1, phenolic acids dominated the nettle leaf water extracts. Among them, chlorogenic and caffeic acids were the most abundant in both extracts. Notably, the sonication assay exhibited greater efficiency in extracting all of the phenolic acids listed in Table 1, especially protocatechuic acid and 4-OH-benzoic acid, with the exception of chlorogenic acid.

We also detected the presence of gallic acid, catechin, epicatechin, quercetin, isorhamnetin, kaempferol, and vanillic acid. However, their concentrations were below the limit of quantification (LOQ) and, therefore, are not presented in Table 1. The mean polyphenol content in the Es was determined to be 45.15 ± 4.65 μg/mL, and in the Em, it was 50.65 ± 4.74 μg/mL.

The extracts contained water-soluble vitamins, of which ascorbic acid was predominant (Table 2). The riboflavin content in the extract obtained using the maceration method was low, whereas the pyridoxine and pantothenic acid concentrations were higher than those in the extract prepared via sonication. Furthermore, the pyridoxine concentration was almost two-fold higher. Sonication resulted in higher extraction of riboflavin and ascorbic acid.

In general, the pantothenic acid, nicotinic acid, and nicotinamide contents in the extracts were comparable, with differences discernible only in the nuances of the analyzed samples.

We compared the microelement mass concentrations in the extracts obtained through maceration and sonication (Table 3). The concentrations of various elements in the extract obtained via sonication, listed from highest to lowest, are as follows: Fe, W, Sr, Mn, Zn, Rb, Ba, Cu, Ni, and Li. In contrast, the maceration procedure resulted in a distinct order of trace element concentrations: Fe, Sr, Mn, W, Zn, Ba, Cu, Li, and Ni. Despite the differences in the amounts of trace elements, both extraction techniques highlighted the nettle extracts as valuable sources of microelements, with a particular emphasis on their richness in Fe.

We also searched for more trace elements, such as V, Co, As, Cr, Pb, Cs, and Bi. However, their mineral mass concentrations were below the detection limit.

### 2.2. Polyphenol Content and Antioxidant Activity of the Nettle Leaf Extracts

We compared the antioxidant activity of the nettle leaf extracts obtained using the maceration and sonication methods. Maceration, being a time-consuming process, necessitated the inclusion of preservatives in the extraction mix, whereas, in the case of sonication, preservatives were added to the end-product; therefore, we were able to prepare additional extracts without preservatives.

**Figure 2:**
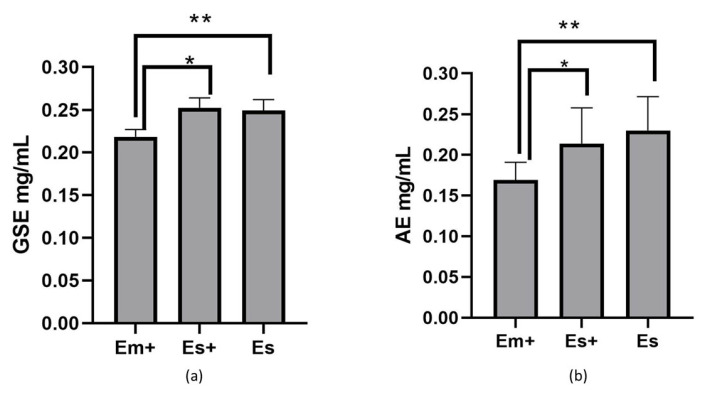
(**a**) Total polyphenol content shown as gallic acid equivalents in mg/mL of nettle extracts. (**b**) Total antioxidant capacity shown as ascorbic acid equivalents in mg/mL of nettle extracts. Abbreviations: Em+ extract obtained via maceration with preservatives; Es+ extract obtained via sonication with preservatives; Es extract obtained via sonication without preservatives. In (**a**), * *p* = 0.0002 vs. Es+ and ** *p* = 0.0005 vs. Es. In (**b**), * *p* = 0.0433 vs. Es+ and ** *p* = 0.0052 vs. Es, according to a one-way ANOVA with Tukey’s multiple comparison test.

The extracts obtained using the maceration method exhibited a lower total polyphenol content (Figure 2a) and a lower antioxidant capacity (Figure 2b).

As shown, the lack of preservatives in the extract Es obtained using the ultrasound method did not change the total antioxidant capacity or polyphenol content compared to extract Es+ obtained using the sonication method when preservatives were added. The calculated GSE mg/g of dry powder for the extract obtained via maceration was 4.2 ± 0.067 GSE mg/g, while that for the extract obtained via sonication with preservatives was 5.1 ± 0.12 GSE mg/g, and that for the extract obtained via sonication without preservatives was 4.98 ± 0.11 GSE mg/g. The calculated AE mg/g of dry powder for extract Em+ was 3.4 ± 0.4, for Es+ was 4.2 ± 0.8 AE mg/g, and for Es was 4.4 ± 0.8 AE mg/g. In this assay, the total antioxidant capacity, expressed as AE mg/g, strongly correlated (r = 0.929) with the total polyphenol content expressed in GAE mg/g.

**Figure 3:**
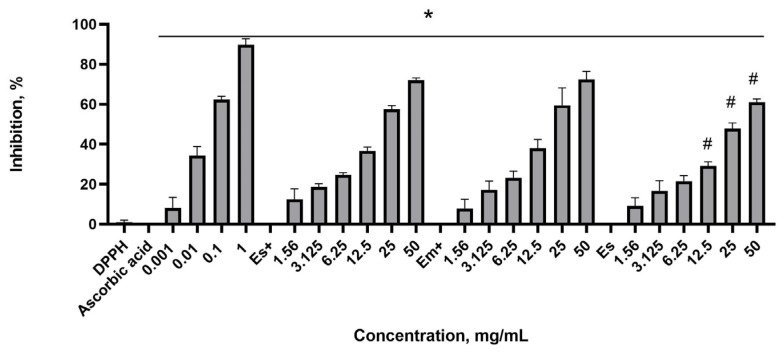
DPPH scavenging effect of ascorbic acid and nettle leaf extracts. Es+ extract obtained via sonication with preservatives; Em+ extract obtained via maceration with preservatives; Es extract obtained via sonication without preservatives. * *p* ≤ 0.05 vs. DPPH concentration when inhibition is 0, according to a one-way ANOVA with Dunnett’s multiple comparison test. ^#^ *p* ≤ 0.05 vs. Es+ and Em+ extracts at corresponding concentrations, according to a one-way ANOVA with Tukey’s multiple comparison test.

In the DPPH scavenging assay, ascorbic acid was used as a positive reference. The data show that ascorbic acid at 1 mg/mL inhibited DPPH completely. The DPPH absorbance at 517 nm was taken as a 100% concentration when inhibition was zero. The nettle leaf extracts inhibited the DPPH concentration at all dilutions (Figure 3). The extract was prepared by extracting 5 g of dry leaf powder per 100 mL of deionized water, resulting in a concentration of 50 mg of dry powder per mL. Meanwhile, a half-diluted (1:1) extract corresponded to 25 mg of dry powder per mL. Further dilutions produced concentrations of 12.5, 6.25, 3.125, and 1.56 mg/mL.

The extract without preservatives (Es) exerted a lower DPPH scavenging effect at concentrations of 50 mg/mL (undiluted extract), 25 mg/mL, and 12.5 mg/mL in comparison to that of the extracts Es+ and Em+ at corresponding dilutions. However, the differences between the more diluted extracts were insignificant. Notably, the effects of the Em+ and Es+ extracts did not differ, and we did not observe the effect of the extraction method in this test. The calculated IC_50_ for Em+ was 21.21 mg/mL, for Es+, it was 29.49 mg/mL, and for Es, it was 32.82 mg/mL. In this assay, the extract inhibition of the DPPH concentration, expressed in IC_50_, strongly correlated (r = 0.918) with the total polyphenol content, expressed in GAE mg/g.

**Figure 4:**
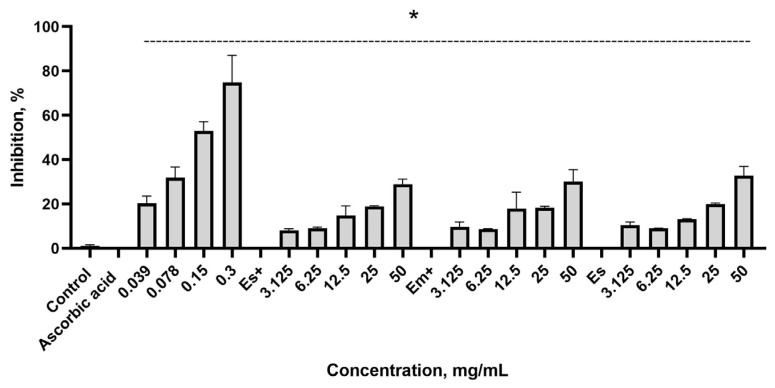
Inhibition of superoxide anion release by ascorbic acid and the nettle leaf extracts. Es+ extract obtained via sonication with preservatives; Em+ extract obtained via maceration with preservatives; Es extract obtained via sonication without preservatives. * *p* ≤ 0.05 vs. superoxide anion concentration when inhibition is 0, according to a one-way ANOVA with Dunnett’s multiple comparison test.

The SOD-mimicking activity of ascorbic acid and the extracts is shown by the inhibition of superoxide anion release (Figure 4). Both ascorbic acid and the extracts exhibited SOD-like activity, with superoxide anion inhibition being statistically significant compared to the control without the inhibitor.

All undiluted nettle leaf extracts showed, at maximum, approximately 30% inhibition, and this effect was similar to that of ascorbic acid at a concentration of 0.078 mg/mL.

### 2.3. Antiglycation Effect of the Nettle Leaf Extracts

**Figure 5:**
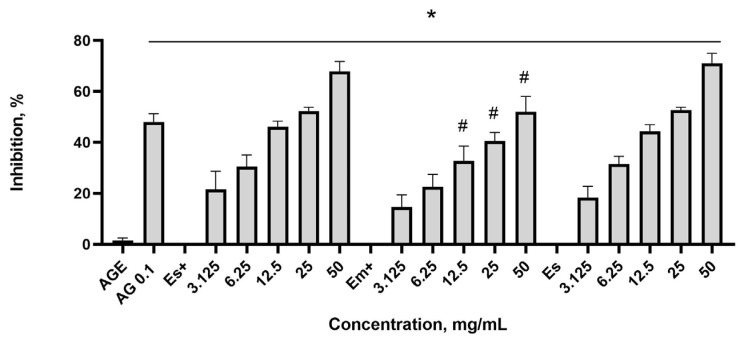
Inhibition of AGE formation by aminoguanidine (AG) and the nettle leaf extracts. Es+ extract obtained via sonication with preservatives; Em+ extract obtained via maceration with preservatives; Es extract obtained via sonication without preservatives. * *p* ≤ 0.05 vs. AGE concentration when inhibition is 0, according to a one-way ANOVA with Dunnett’s multiple comparison test. ^#^ *p* ≤ 0.05 Em+ vs. Es+ and Es extracts at corresponding concentrations, according to a one-way ANOVA with Tukey’s multiple comparison test.

The formation of advanced glycation end (AGE) products in vitro was investigated by subjecting bovine serum albumin (BSA) to a reaction with methylglyoxal (MGO) with and without nettle extracts, followed by a one-week incubation at 37 °C in the absence of light. The fluorescence of AGEs was quantified, and the inhibitory effects of aminoguanidine, serving as a positive reference, and the nettle leaf extracts were determined based on the changes in relative fluorescence units (RFUs) and then recalculated as inhibition percentage (Figure 5).

All nettle extracts inhibited AGE formation at all assessed dilutions compared to the effect with the AGE control. However, when comparing the mean of each column with the mean of every other column, it became evident that the extract obtained via maceration showed lower AGE inhibition potency than the extracts obtained using the ultrasound technique. The undiluted extracts obtained via sonication with and without preservatives showed a maximal effect of approximately 70% inhibition, whereas the extract obtained using the maceration method exhibited approximately 50% inhibition. The difference was also seen at dilutions of 25 and 12.5 mg/mL. Aminoguanidine at a concentration of 0.1 mg/mL exhibited a significant inhibitory effect on AGE formation, resulting in an approximate 50% reduction.

### 2.4. Hepatoprotective Effects of the Nettle Leaf Extracts In Vitro

**Figure 6:**
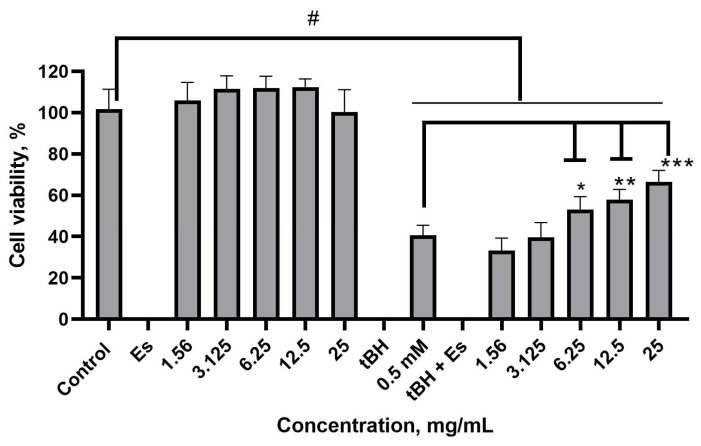
Effects of the nettle leaf extracts on cell viability. ^#^ *p* ≤ 0.05 vs. the control (HepG2 cells without the extracts), according to a one-way ANOVA with Dunnett’s multiple comparison test. * *p* = 0.0298, ** *p* = 0.0007, *** *p* < 0.0001 vs. the tBH control, according to a one-way ANOVA with Tukey’s multiple comparison test.

To ensure pH compatibility with the cellular environment, we chose the preservative-free extract obtained using the ultrasound technique to investigate hepatoprotective effects. The hepatoprotective effects of the nettle extracts on cell viability in an oxidative stress environment were investigated using tert-butyl hydroperoxide (tBH)-induced oxidative stress. The results indicate that even at the highest dilution employed (Figure 6), the extract per se did not significantly affect cell viability compared to the control cells (HepG2 cells without the extracts). Cell viability was expressed as a percentage relative to the untreated controls. Oxidative stress led to a reduction in cell viability of approximately 40%.

At concentrations of 6.25 to 25.0 mg/mL, the extract showed an increase in HepG2 cell viability compared to the tBH control. However, complete cell recovery from oxidative stress was not reached compared to the control. The IC_50_ value was calculated as 6.314 mg/mL.

**Figure 7:**
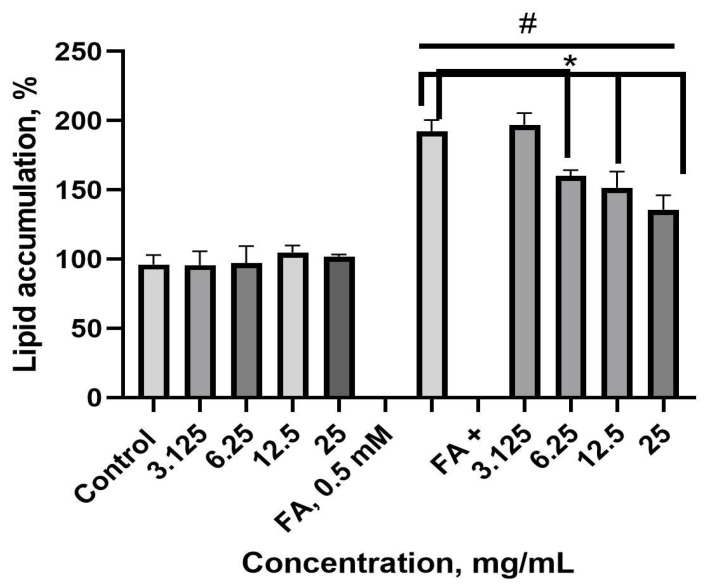
Effects of the nettle leaf extract on lipid accumulation in HepG2 cells. ^#^ *p* ≤ 0.05 vs. the control (untreated HepG2 cells), according to a one-way ANOVA with Dunnett’s multiple comparison test. * *p* < 0.0001 vs. the fatty acid mix (FA) control, according to a one-way ANOVA with Tukey’s multiple comparison test.

To create a liver steatosis model, HepG2 cells were cultured in a lipid overload-conditioned medium. Control cells were grown in a medium without a fatty acid mix (0.5 mM, 2:1 mix of oleic acid and palmitic acid, respectively). The effect of the nettle leaf extract on lipid accumulation in the cells was observed using Nile red fluorescent staining, with the results expressed as relative fluorescence units (RFUs) in percentages. As shown in Figure 7, the extract per se did not change the lipid level in the control HepG2 cells, whereas in the lipid-conditioned medium, it decreased lipid accumulation.

Fatty acid overload increased the lipid levels approximately two-fold compared to the control cell lipid levels. The nettle extract, at concentrations ranging from 6.25 to 25 mg/mL, prevented lipid accumulation compared to the fatty acid control. However, they did not provide complete protection, as lipid accumulation remained elevated compared to the control cell levels. The IC_50_ value for the reduced lipid levels was calculated to be 5.56 mg/mL.

### 2.5. Effects of the Nettle Leaf Extracts on Oxygen Flux in Different OXPHOS States in the Fatty Acid Oxidation Pathway

**Figure 8:**
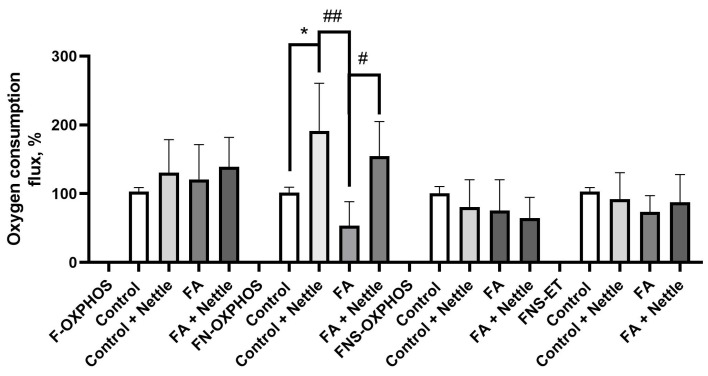
Measurement of oxygen consumption flux using high-resolution respirometry. Representative graphs depict fatty acid oxidation (F-OXPHOS), FN-OXPHOS (complex I (CI) activity), FNS-OXPHOS (complex I and complex II (CI and CII) activity), FNS-ET (CI and CII and electron transfer (ET) activity) activity in HepG2 cells treated with a nettle extract and/or fatty acid mixture (0.5 mM, 2:1 mix of oleic acid and palmitic acid, respectively). Data are expressed as a percentage from assessed pmol O_2_/(s × 10^6^ cells). Statistical analysis was performed using a one-way ANOVA followed by a post hoc Tukey’s test. Data are presented as means (percentage of control data) ± SDs. * *p* = 0.0018 vs. the control; ^#^ *p* = 0.0108 vs. FA; ^##^ *p* < 0.0001 vs. FA. Experiments were repeated 12–14 times.

High-resolution respirometry was used to detect the oxygen flux in different oxidative phosphorylation (OXPHOS) states in the fatty acid oxidation (FAO) pathway. The HepG2 cells treated with the nettle extracts at a concentration of 6.25 mg/mL—close to the IC_50_ calculated in earlier oxidative stress and lipid accumulation assays (control + nettle, Figure 8)—exhibited significantly higher oxygen consumption in the FN-OXPHOS state (associated with complex I and fatty acid oxidation activity) compared to the control group baseline (set at 100%). When a fatty acid mixture was added to the HepG2 cells (FA group), the FN-OXPHOS capacity decreased compared to the control group. However, the reduction was not statistically significant compared to the control oxygen flux level but was significantly lower than that in the control + nettle group. Notably, simultaneous treatment of cells with a nettle extract and fatty acid mixture (FA + nettle) significantly increased complex I activity compared to the FA group.

No statistically significant differences were observed in the F-OXPHOS, FNS-OXPHOS, or FNS-ET assays. The results indicate that oxygen flux in the lipid + nettle group was normalized to the control group level.

## 3. Discussion

The latest reviews have confirmed the value of nettle extracts in the development of food, cosmetic products, and nutritional supplements. The scientific validation of these various uses is substantiated by the presence of bioactive compounds in stinging nettles, such as phenolic compounds, sterols, fatty acids, alkaloids, terpenoids, flavonoids, and lignans [1,2,3,4,5,6,7,8,11,14,18,19,20,21,22,23,24].

In our study, we used aqueous extracts obtained using the maceration and ultrasound-assisted methods. Many polyphenols are insoluble or poorly soluble in water, which may explain why we mainly found phenolic acids (chlorogenic, caffeic, protocatechuic, 4-OH-benzoic, o-coumaric, ferulic, and sinapic acids) in the phytochemical compositions of the extracts. The dominant compounds in the extracts were chlorogenic acid, caffeic acid, and rutin. It should be noted that these compounds themselves exhibited effects characteristic of the whole extract. As reviewed in [25,26], chlorogenic acid is an important and biologically active dietary polyphenol, demonstrating antioxidant, free radical-scavenging, anti-inflammatory, antipyretic, neuroprotective, and CNS-stimulating activities. It is also associated with a reduced risk of metabolic syndrome through hepatoprotective, cardioprotective, anti-obesity, and anti-hypertension effects. In addition to caffeic acid’s antioxidant and anti-inflammatory effects, the role of caffeic acid in the prevention and treatment of metabolic syndrome has recently been highlighted [27].

Maceration resulted in a higher extraction of chlorogenic acid and rutin from the nettle leaves, whereas the content of caffeic acid did not differ in the extracts obtained using the maceration and sonication methods. The finding of rutin increases the value of the extracts. The effects of rutin have been highlighted [28], showcasing a broad spectrum of pharmacological activities, including antioxidant, cytoprotective, vasoprotective, anticarcinogenic, neuroprotective, and cardioprotective effects.

We also detected the presence of gallic acid, catechin, epicatechin, quercetin, isorhamnetin, kaempferol, and vanillic acid in both extracts. Despite their low concentrations, their cumulative effect can be significant.

In the future, modifications could be introduced into the extraction process to extract more polyphenols. A study published in 2022 [29] found that based on mathematical models, the best nettle leaf extraction conditions were ultrasound treatment with water as the solvent and an extraction time of 3.15 h, obtaining varying values from 10 to approximately 27 GAE mg/g dried nettle leaves. The extraction result depended on ultrasound, extraction time, temperature, and stirring. In a Lithuanian study [30], the polyphenol content was calculated to be around 15.3 GAE mg/g. These Lithuanian researchers concluded that the most effective extraction conditions for total polyphenol content were extraction at 70 °C for 40 min. In our experiments, 5 g of dry leaf powder was extracted in 100 mL of water. We calculated approximately 4.2 to 5.1 GAE mg/g of dry nettle leaf powder. It is widely acknowledged that the composition and quantity of chemical compounds are influenced by climatic zones and growing conditions. Given the expansive range of climatic zones where nettles grow, significant variations in extract compositions are expected [11,23,24,30]. For instance, nettle leaf samples collected from continental areas exhibit higher levels of polyphenols, whereas those from coastal regions are notably enriched with pigments [23]. Our study was conducted on nettle specimens cultivated in Latvia, a country situated in the Northern Europe temperate climate zone.

As noted in [1,2,20,23], stinging nettle leaves contain minerals, such as calcium, potassium, magnesium, phosphorus, iron, sulfur, zinc, manganese, copper, and nickel. Numerous authors have investigated the presence of microelements in nettle root and leaf extracts; however, different extraction techniques are often employed, leading to varying concentrations of trace elements. We observed that both extraction methods successfully yielded microelements, with iron registering the highest levels, consistent with findings from other authors. Moreover, our analysis did not detect harmful trace elements such as Pb or As. This result confirms the quality conditions for cultivating nettles on a farm certified for growing medicinal plants, where the nettle leaves were collected and the extracts prepared.

In this nettle extract study, we found water-soluble vitamins, such as riboflavin, pantothenic acid, ascorbic acid, pyridoxine, nicotinic acid, and nicotinamide. Sonication resulted in a higher extraction of riboflavin and ascorbic acid, whereas maceration provided higher concentrations of pyridoxine and pantothenic acid. The ascorbic acid concentration was the highest in both extracts. The role of water-soluble vitamins in the antioxidant capacity of plant extracts has been discussed from various angles. Computational investigation studies [31] have suggested that ascorbic acid possesses the highest antioxidant effect in the aqueous environment. Some studies have pointed to other vitamins, including pyridoxine and riboflavin, as classical antioxidants, playing vital roles in defending the body against free radicals by potentiating enzyme antioxidants, acting as co-enzymes in their reduced physiological forms, or by directly attacking free radicals [32].

Reviewing the frequency of the use of antioxidant in vitro methods, the authors of [33,34,35] concluded that each method has advantages and disadvantages. We had no doubts that polyphenol- and vitamin-containing nettle extracts would possess antioxidant effects. Therefore, for comparison of the extracts’ antioxidant capacity, we chose a limited number of methods, such as TAC, DPPH, and SOD-mimicking assays. As expected, we observed antioxidant effects regardless of the antioxidant assay. However, we observed that the extracts obtained using the maceration method exhibited a lower total antioxidant capacity compared to the effects of the extracts obtained using the sonication method. DPPH assays are one of the most popular colorimetric assays for estimating the radical scavenging capacity of extracts or individual compounds. This method provides a screening of the general activity of antioxidants. In earlier published studies [29], nettle water extracts obtained via ultrasound extraction using stirring extraction, water as the solvent, and 3 h of extraction time exerted a strong antioxidant effect in the DPPH assay (91.08%). In our experiments, the maximal scavenging effect of the undiluted extracts was around 70%. We noticed that the extract obtained via sonication without preservatives exerted a lower DPPH scavenging effect than that of the extracts with preservatives at corresponding dilutions. We hypothesize that the presence of citric acid in the added preservatives may have potentiated the antioxidant capacity of the extracts. The literature data support this assumption. Indeed, it was shown in vitro, in animal and clinical studies, that citric acid is associated with the antioxidant and anti-inflammatory action in cells [36,37,38]. Citric acid has demonstrated a significant antioxidant effect in DPPH assays [37,38].

In the SOD-mimicking assay, we observed a maximum 30% inhibition of superoxide anion release using undiluted nettle leaf extracts. We found that this effect was like the effect of ascorbic acid at a concentration of 0.078 mg/mL. The SOD-mimicking potential of water nettle extracts has also been investigated by other researchers using both in vitro and in vivo assays, yielding divergent conclusions. For example, Turkish researchers found a strong superoxide anion radical scavenging effect of water extracts of nettle aerial parts [39]. In contrast, in in vivo experiments, when C57Bl6 mice were treated for 15 days with a nettle extract at a dose of 40 mg of total polyphenols in the extract per kg of mouse body weight, a statistically significant effect on the SOD level in the liver of the mice was not observed [40].

The potential of dietary constituents, enriched foods, or supplements containing natural bioactive molecules to inhibit or stop AGE development has been repeatedly highlighted [41,42,43]. The accumulation of AGEs has been suggested to be a pathogenic mechanism of many diseases, including diabetes, atherosclerosis, neuropathy, retinopathy, nephropathy, aging, and chronic renal disease. In our study, nettle extracts, regardless of the method of extraction and the presence of preservatives, inhibited AGE formation at all dilutions assessed compared to the AGE control. However, when comparing the effect of one extract versus the effect of another extract, we observed that the extract obtained via maceration showed lower AGE inhibition potency than the extracts obtained via sonication.

It is worth noting that water extracts have a limited shelf life due to the potential development of mold and other bacterial contaminants, as well as the fermentation process. Therefore, manufacturers often incorporate various preservatives, particularly in the maceration method, where they should be introduced at the outset. The question of whether to add preservatives to water extracts is debatable because plant extracts can be lyophilized and stored as a dry powder until use without preservatives. Furthermore, several scientific studies have acknowledged the antimicrobial and antioxidant properties of plant polyphenols, allowing for the use of polyphenol-rich plant extracts as preservatives in food, such as meat [44,45,46,47,48]. However, in these cases, dry or lyophilized extracts were used, which is not an option for liquid extracts. The preservative properties of natural substances have received significant interest in recent years, including the benefits of water or water–ethanol plant extracts as natural additives for food applications [47,48]. Some natural compounds, including polyphenols, have been tested and have shown positive results against food-related toxins [49]. In this study, we used nettle leaf extracts with citric acid, sodium benzoate, and potassium sorbate added as preservatives. These compounds were utilized as preservatives for food, such as during the storage of orange-fleshed sweet potato puree [50]. Here, we did not examine the bacterial contamination of the extracts; therefore, we cannot confirm the protective effectiveness of the used preservatives.

In cell assays where an acidic pH is not applicable, we opted for an extract without preservatives. The obtained data demonstrated that this extract per se did not influence HepG2 cell viability, whereas, in an oxidative stress model, the nettle extracts significantly improved cell survival, demonstrating antioxidant defense.

The finding that free fatty acid-overloaded human hepatocytes and HepG2 cells reached similar levels of maximal intracellular lipid accumulation, which were very close to that determined in hepatocytes from human steatotic livers, supports the use of HepG2 cells for in vitro steatosis modeling [9,10,51]. Notably, a ratio of oleic acid and palmitic acid of 2:1 was suggested as the best variant for in vitro models of hepatocellular steatosis with a lipid overload profile, excluding cytotoxic/apoptotic effects [9,10]. Therefore, this approach was used in our experiments to create a fatty liver model in the HepG2 cell line. Our data showed that the nettle extracts per se did not increase the lipid level in the cells, whereas they inhibited free fatty acid mix-induced lipid accumulation. The obtained data are consistent with in vivo experiments. For example, nettle extract consumption in mice boosted lipid metabolism by modulating selected liver cell transcriptional factors and histone deacetylase [40]. Moreover, a similarity between nettles’ effects and the action of statins has been observed [6]. It was clarified that the antihyperlipidemic properties of nettles are mediated by the inhibition of the enzyme 3-hydroxy-3-methylglutaryl coenzyme A reductase in rats and the amelioration of lipid peroxidation via antioxidant effects. NAFLD is primarily caused by four mechanisms: excessive lipid uptake, de novo lipogenesis, β-oxidation of fatty acids, and the export of hepatic lipids [7]. Peroxisome proliferator-activated receptor-α (PPAR-α) is one of the primary regulators of fatty acid oxidation in mitochondria, peroxisomes, and cytochromes [7]. In patients with type 2 diabetes mellitus, nettle extract consumption resulted in PPAR-α upregulation, along with increased fatty acid oxidation in the liver [52]. Another patient study revealed that several herbal preparations, including ethanol nettle extracts, activated human PPAR receptors. These extracts acted similarly to clinically used lipid-lowering drugs, such as synthetic PPAR-α (fibrates) and PPAR-γ (glitazones) activators, lowering serum LDL levels [53].

The high-resolution respirometry method, being the state-of-the-art approach in mitochondria and cell research, has been developed for various cell lines, including the HepG2 cell line [54]. We employed this sensitive technique to evaluate the influence of nettle extracts on state-dependent mitochondrial activity through oxygen consumption measurements. This method allowed us to precisely assess respiration dynamics at complexes I and II of the electron transport system (ETS) and compare mitochondrial functionality in permeabilized HepG2 cells treated with lipids and/or nettle extracts. Our results using the HepG2 cell model of NAFLD showed an apparent decrease in oxygen consumption caused by the fatty acid mixture compared to the control cells. However, despite many repetitions of the experiments, the large fluctuations in the results did not lead to a statistically confirmed reduction. The complexity of mitochondrial studies in lipid-overloaded hepatic cells has been highlighted by recent findings of two distinct populations of mitochondria in male Wistar rats on an ad libitum diet: cytoplasmic mitochondria and lipid droplet-associated mitochondria. These studies revealed that lipid droplet mitochondria exhibited higher fatty acid oxidation, whereas cytoplasmic mitochondria were associated with higher respiration capacity, thus demonstrating a distinct bioenergetic pattern. The authors suggested the importance of functional segregation of mitochondria [55].

In our study, the nettle extracts increased the FN-OXPHOS activity and mitigated a fatty acid-induced decrease in FN-OXYPHOS activity. The FN-OXPHOS capacity reflects the mitochondrial respiratory function at the FN junction (also known as the FN pathway), indicating changes in complex I activity during the OXPHOS state.

We did not find any literature on the effects of nettle leaf extracts on liver mitochondria or fatty acid oxidation pathways in permeabilized HepG2 cells. Our data are the first to demonstrate the significance of mitochondria-related respiration effects in the mechanism of nettle’s hepatoprotective properties.

## 4. Materials and Methods

### 4.1. Extraction Methods

*Urtica dioica* L. leaves were collected from a farm certified for growing medicinal plants called “Kurmīši” in Latvia, and the extract preparation was carried out by Rasa Botanicals Ltd. The nettle leaves were harvested from late July to August. The collected nettle leaves were washed and placed on sieves to dry. After two days, the leaves were placed in a ZYPROMAX plant dryer (Zyle, Kaunas, Lithuania) for 10 h at 40 °C. The dried leaves were cooled and ground in a TP2 crushing/grinding machine (Tecnolab, Bassano del Grappa, Italy). The obtained particle size was, on average, 2 mm in diameter. Then, the ground leaf powder was weighed (Kern-Sohn scales, Kern, Germany), added to water at proportions of 5 g of leaf powder and 100 mL of deionized water, mixed in a Witeg HS-120A mixer (Witeg, Wertheim, Germany), and then left to stand for the 12 h at room temperature (RT). Following this, sonication was performed using the UIP1000hd T apparatus (Hielscher, Teltow, Germany) with a power of 100 W/g, while the temperature did not exceed 40 °C. After sonication, the samples were left to stand for 1 h at RT. The samples were filtered through filter paper with a pore size of 8–15 μm (Watman Filter paper grade 1, Watman, FL, USA) using an ME 1C 721,110 vacuum pump (Vacuubrand, Wertheim, Germany), a Bichner funnel (Drifton, Hvidovre, Denmark), and a Bunsen flask (Kavalierglass Co., Prague, Czech Republic). As preservatives, 0.5% potassium sorbate and sodium benzoate were added to the filtered extract, and food-grade citric acid was added to adjust the pH of the extract to be between 3.5 and 3.8. Another extract sample was prepared without preservatives.

**Figure 9:**
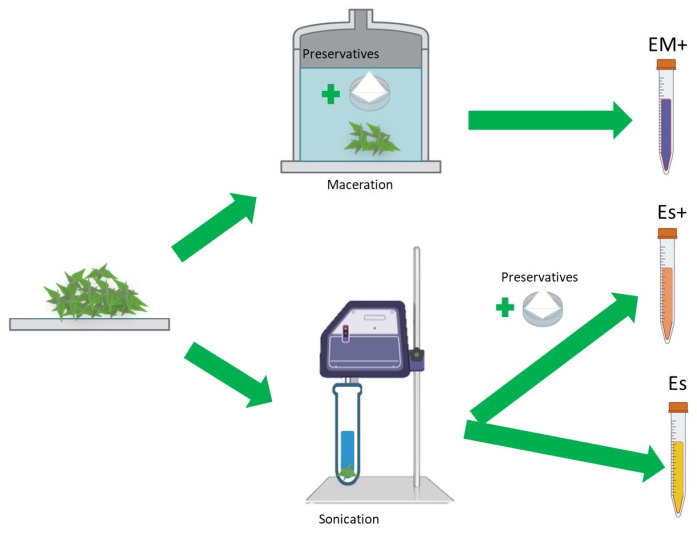
Extract preparation. Abbreviations: EM+: extract obtained via maceration with preservatives; Es+: extract obtained via sonication with preservatives; Es: extract obtained via sonication without preservatives.

Using the maceration method, dried leaf powder was prepared as described before, but preservatives and citric acid were added to deionized water. The proportions of dry powder and deionized water were the same—5 g per 100 mL. Maceration was carried out in a dark glass container in a dark room at RT for 30 days. Afterward, the obtained extract was filtered as described before (Figure 9).

The finished extracts were packaged in dark glass bottles.

### 4.2. Analysis of Polyphenols

Reference compounds of the highest grade were purchased from Sigma-Aldrich (Labochema Latvia, Riga, Latvia). Acetonitrile (Fisher Chemical, Pitsburg, PA, USA) and formic acid (Thermo Scientific, Waltham, FL, USA), both of LC/MS grade, were used. Individual stock solutions of the compounds were prepared in 60% MeOH at a concentration of 50 mg per L, except for (+)–catechin, (-)–epicatechin, isorhamnetin, myricetin, naringenin, and quercetin, which were dissolved in acidified MeOH (1% formic acid) to increase their stability in the solution. The limit of detection (LOD) and the limit of quantification (LOQ) were calculated for each compound. These parameters were calculated at signal-to-noise (S/N) = 3.3 and 10, using the ratio between the standard deviation of regression by the slope of a calibration curve. The Waters Acquity UPLC^TM^ (Waters, Milford, MA, USA) system consisted of a quaternary solvent manager, a sample manager (FTN), a column heated compartment photodiode array detector (PDA eλ), and a mass spectrometer (Waters Xevo TQD, Zspray^TM^). The method for analysis of 19 polyphenolics used an Acquity UPLC BEH™ C18 column (1.7 μm, 2.1 × 150 mm) at 35 °C. Masslynx 4.2 software (Waters Corporation) was used for data acquisition and instrument control. The source parameters of MS, such as capillary voltage, cone voltage, source temperature, desolvation temperature, cone gas flow, desolvation gas flow, and collision gas, were set at 3.0/3.5 kV (ES+/ES−), 70 V, 150 °C, 380 °C, 40 L/h, 800 L/h, and 3.5 × 10^−3^ mBar (argon), respectively. For full-scan ESI–MS/MS, the spectra scan range was *m*/*z* = 80–1000 at unit resolution. The cone voltage and collision energy were optimized for each compound to achieve specific and stable multiple reaction monitoring (MRM) transitions. The ion pairs of caffeic acid, catechin, chlorogenic acid, o-coumaric acid, *p*-coumaric acid, epicatechin, ferulic acid, 4-hydroxybenzoic acid, isorhamnetin, kaempferol, myricetin, naringenin, protocatechuic acid, quercetin hydrate, resveratrol, rutin hydrate, sinapic acid, syringic acid, and vanillic acid were performed with transitions of *m*/*z* = 179.0 → 134.9, 291.0 → 165.0, 353.0 → 190.9, 163.0 → 118.9, 163.0 → 118.9, 291.0 → 139.0, 193.0 → 133.9, 137.2 → 92.9, 317.2 → 301.9, 286.7 → 152.9, 318.9 → 152.9, 272.9 → 152.9, 152.9 → 109.1, 303.0 → 229.0, 227.0 → 143.0, 611.0 → 303.0, 225.0 → 207.0, 199.0 → 139.8, and 169.0 → 92.9, respectively, and they were selected as quantification traces by MRM mode.

The mobile phase was composed of 10 mM ammonium formate in water acidified with 0.3% formic acid (A) and 0.3% formic acid in acetonitrile (B), which was used in the following gradient elution mode: 0–2 min, 3% B; 2–12 min, 3–13% B; 12–18 min, 13–33% B; 18–24 min, 33–48% B; 24–30 min, and then returned to the initial composition in 5 min. The injection volume was 2.0 μL.

### 4.3. Analysis of Water-Soluble Vitamins

Reference compounds of the highest grade were purchased from Sigma-Aldrich (Labochema Latvia, Latvia). Methanol (Fisher Chemical) and formic acid (Thermo Scientific) of LC/MS grade were used. A UPLC ESI-MS method, coupled with multiple reaction monitoring (MRM) and UV detection, was introduced for water-soluble vitamin analysis. The water-soluble vitamin MS conditions, including MRM acquisition mode and monitored ion transition, were adjusted according to Waters^TM^ application notes [56]. The LC conditions comprised an Acquity UPLC HSS T3 C18 column (1.8 µm, 2.1 × 150 mm) at 40 °C, a solvent system consisting of 10 mM ammonium formate and 0.1% formic acid (A) and 10 mM ammonium formate and 1% formic acid in methanol (B), and an autosampler system that thermostated samples at 4 °C and carried out injections of 2 μL in loop. The mobile phase was used in the following gradient elution mode: 0–1 min, 1–8% B; 1–4 min, 8–35% B; 4–10 min, 13–33% B; 35 min, and within 5 min was returned to the initial composition at a total flow rate of 0.25 mL min^−1^. The water-soluble vitamin MS chromatography conditions, including MRM acquisition mode, mobile phase composition, and gradient modes, were adjusted according to Waters^TM^ application notes [56]. Stock solutions of pantothenic acid (B5), ascorbic acid (C), pyridoxine (B6), nicotinic acid (B3), and nicotinamide (B3′) were prepared using deionized water. For the vitamins riboflavin (B2) and folic acid (B9), stock solutions were prepared in 100 mM ammonium formate.

All chromatograms are shown in the Supplementary Materials.

### 4.4. Analysis of Mineral Mass Concentrations

First, 10 mL of the nettle extracts earmarked for analysis were transferred into 25 mL glass test tubes and placed in a water bath set at 80 °C, allowing the solvent to gradually evaporate under a gentle stream of nitrogen. Subsequently, 3 mL of concentrated nitric acid was added to the dry residue of the nettle extracts. The test tubes were again placed in a water bath at 80 °C and heated until all remnants of the dry nettle extracts dissolved and the intensive release of nitrogen (IV) oxide stopped. The resulting nettle extracts in nitric acid were quantitatively transferred from the test tubes to volumetric flasks and then diluted to a final volume of 50 mL using deionized water.

To construct a calibration curve, standard solutions with specific mass concentrations (ranging from 1 to 1000 μg/L) of the elements to be determined were prepared. The signal intensity of these chemical elements in the calibration solutions was measured using an Agilent ICP-MS 8900 Triple Quadrupole inductively coupled plasma mass spectrometer (Santa Clara, CA, USA). The ICP-MS parameters were an RF power of 1550 W, a sampling depth of 8 mm, a plasma gas flow rate of 15.0 L ∙ min^−1^, a nebulizer gas flow rate of 0.90 L ∙ min^−1^, and a He cell gas flow of 5.0 L ∙ min^−1^. A calibration graph was then generated by plotting the measured signal intensity of the chemical elements on the *y*-axis, while the mass concentrations of the elements were plotted on the *x*-axis. Subsequently, inductively coupled plasma mass spectrometry (ICP-MS) was employed to measure the signal intensity of the chemical elements in both the nettle extracts and the “blank” sample, consisting solely of ultra-pure nitric acid and deionized water. Utilizing the intensity values of the signals from the chemical elements in the nettle extracts, obtained through ICP-MS, previously established calibration graphs were employed to calculate the mass concentration of the chemical elements in the undiluted nettle extracts.

### 4.5. Determination of the Antioxidant Activities of the Extracts Using Total Antioxidant Capacity (TAC) Assays, the Total Polyphenol Folin–Ciocalteu Colorimetric Method, and 2,2-diphenyl-1-picrylhydrazyl (DPPH) and Superoxide Dismutase (SOD) Assays

All reagents were purchased from the Merck group, including Sigma-Aldrich (Merck, Darmstadt, Germany), while the materials (tubes, microplates, and pipettes) were obtained from Sarstedt (Nümbrecht, Germany).

The total antioxidant capacity (TAC) of the extracts was determined using the green phosphomolybdenum complex method described by others and used by us previously [35,57]. The extract (5 g of dry leaf powder extracted with 100 mL of water) was considered 100% or undiluted, and a series of dilutions were prepared. Accordingly, the dry leaf powder concentrations in the dilutions were 50 mg/mL, 25 mg/mL, 12.5 mg/mL, 6.25 mg/mL, 3.125 mg/mL, and so on. Ascorbic acid solutions were prepared at concentrations of 1, 0.5, 0.25, 0.125, 0.0625, 0.03125, and 0.0156 mg/mL. Deionized water and water with 0.5% sodium benzoate, potassium sorbate, and citric acid were used as blank controls. Then, 0.1 mL of each extract or ascorbic acid at different concentrations was mixed with 0.9 mL of the reagent solution, consisting of 0.6 M sulfuric acid, 28 mM sodium phosphate, and 4 mM ammonium molybdate in deionized water in Eppendorf tubes. The tubes were incubated in a dry thermal bath at 95 °C for 90 min. After cooling to RT, 150 µL of the samples in triplicates were pipetted into 96-well microplates, and absorbance was measured at 695 nm against a blank (water or water with preservatives) using an Infinite 200 PRO plate reader and i-control software (Tecan Trading AG, Männedorf, Switzerland). An ascorbic acid standard curve was used to calculate ascorbic acid equivalents per mL of an extract (AE mg/mL) or AE mg/g of dry leaf powder.

Total polyphenols were quantified using the Folin–Ciocalteu colorimetric microplate method [58] with some modifications. Folin–Ciocalteu reagent was diluted with water at a 1:10 ratio. Gallic acid solutions were prepared at concentrations of 1, 0.5, 0.25, 0.125, 0.0625, 0.03125, 0.0156, and 0.0078 mg/mL. The reaction mixture consisted of 20 µL of undiluted or diluted extracts, gallic acid samples, or a blank and 50 µL of Folin–Ciocalteu reagent per well in a 96-well microplate in triplicates. After 5 min, 80 µL of 7.5% sodium bicarbonate solution was added. Measurements of the absorbance were carried out at 750 nm using an Infinite 200 PRO plate reader and i-control software after a 20–30 min incubation period at RT in the dark. A gallic acid standard curve was used to calculate gallic acid equivalents per mL (GAE mg/mL) or GAE mg/g of dry leaf powder.

For the DPPH radical scavenging microplate assay, 20 µL of the undiluted or diluted extract samples was mixed with 180 µL of 0.5 mM DPPH in methanol in a 96-well plate. Appropriate blanks (methanol, water, and water with preservatives) and standards (ascorbic acid) were also run. The plate was kept in the dark for 30 min. The absorbance of the samples was measured at 517 nm using an Infinite 200 PRO plate reader and i-control software (Tecan Trading AG, Switzerland). Ascorbic acid samples were tested over concentrations ranging from 0.001 to 1 mg/mL. This method closely followed the procedures used by others and by us previously [57,59]. DPPH scavenging effect (%) = [(A_1_ − A_0_)/A_1_] × 100, where A_1_ is the absorbance of the control (DPPH solution without sample) at 517 and A_0_ is the absorbance at 517 of the sample at different concentrations with DPPH.

Superoxide dismutase activity was determined using a kit (Product No 19160) purchased from Sigma-Aldrich (St. Louis, MO, USA), and measurements were carried out according to the kit manufacturer’s instructions. The SOD-mimicking activity of the extracts was determined as the superoxide anion inhibition activity and quantified by measuring the decrease in the superoxide anion concentration, which is proportional to the WST-1 formazan color development at 440 nm. Ascorbic acid was used as a positive reference substance. The absorbance of the samples was measured at 440 nm using an Infinite 200 PRO plate reader and i-control software (Tecan Trading AG, Männedorf, Switzerland).

### 4.6. AGE Formation in Bovine Serum Albumin (BSA) and Methylglyoxal (MGO) Reaction

In the BSA-MGO reaction, the formed AGE was detected using published assays [43,60] with modifications. First, 200 µL of BSA (10 mg/mL), MGO (5 mM), and undiluted or diluted extracts were pipetted into Eppendorf tubes in triplicates. As a positive control, aminoguanidine at a concentration of 0.1 mg/mL was used, whereas the blank controls were water and water with preservatives, as well as an AGE formation control with phosphate buffer at pH 7.0 instead of the extracts. The samples were incubated for 7 days at 37 °C and, after that, cooled at RT. Subsequently, 150 µL from each sample was transferred in triplicate to black microplates. Intrinsic fluorescence was measured with excitation and emission wavelengths of 360 and 435 nm, respectively. The fluorescence of the samples was measured using an Infinite 200 PRO plate reader and i-control software (Tecan Trading AG).

### 4.7. Oxidative Stress and Steatosis Modeling in a Human Hepatic Cell Line

#### 4.7.1. Cell Culturing

The human hepatocarcinoma cell line HepG2 (ATCC HB-8065) was obtained from the American Tissue Cell Culture (ATCC) bank (Manassas, VA, USA). Cells were grown in Dulbecco’s modified Eagle’s medium supplemented with 10% heat-inactivated fetal bovine serum (FBS) and 1% antibiotic mix (penicillin 100 units/mL and streptomycin sulphate 100 µg/mL). Cell culturing media, FBS, and other reagents were purchased from Sigma-Aldrich (St. Louis, MO, USA). The cells were cultured at 37 °C in a humidified atmosphere of 5% CO_2_ until confluence. For the experiments, the cells, at a quantity of 2 × 10^4^ per well, were seeded in 96-well microplates and allowed to adhere for 24 h.

#### 4.7.2. Cell Viability in the Oxidative Stress Assay

HepG2 cells at a density of approximately 2 × 10^4^ cells per well were plated into 96-well plates and, after 24 h, treated with various dilutions of nettle leaf extract obtained using the ultrasound method without preservatives for 24 h. The cell viability was evaluated following the CCK-8 kit manufacturer’s instructions (CCK-8 kit, Sigma 96992–500TESTS-F, Sigma-Aldrich, St. Louis, MO, USA). The absorbance was measured at 450 nm using an Infinite 200 PRO plate reader and i-control software (Tecan Trading AG, Switzerland). The absorbance of untreated cell samples was taken as 100% viability. The experiment was performed in duplicate and repeated three times. Cell viability was expressed as a percentage relative to the untreated controls. Consequently, noncytotoxic concentrations of the extracts were selected for hepatoprotective studies.

The hepatoprotective activity of the extracts was evaluated against *tert-*butyl hydroperoxide (tBH)-induced cell oxidative stress. The HepG2 cells were pretreated with the extract at varying concentrations for one hour and then exposed to 0.5 mM *t*BH for 2.5 h in the presence/absence of the extract. Cell viabilities were monitored using a CCK-8 assay. Cell viability is expressed as a percentage relative to the untreated controls.

#### 4.7.3. Liver Steatosis Assay

HepG2 cells were allowed to attach to black 96-well microplates for at least 24 h before initiating lipid overloading. Lipid overloading was carried out for 24 h in the presence of oleate/palmitate at a ratio of 2:1 (0.5 mM) in fatty acid-free 10% BSA. The assay used was that of previously published methods [10,61,62]. The lipid mixture in the absence and presence of the test extract obtained via sonication without preservatives at different dilutions was incubated for 24 h at 37 °C and 5% CO_2_. Then, the culture medium was gently removed and the cells were washed with sodium chloride buffered phosphate buffer (PBS). Nile red staining was carried out according to the kit manufacturer’s instructions (ab228553, Nile Red Staining Kit, Abcam, Cambridge, UK). The lipid droplets in the cells stained with Nile red were observed under a microscope and quantified using a fluorescence plate reader with excitation at 550 nm and emission at 640 nm. The fluorescence of the samples was measured using an Infinite 200 PRO plate reader and i-control software (Tecan Trading AG).

In the mitochondria functionality assay, a 0.5 mM fatty acid mixture with and without the nettle extract at a concentration of 6.25 mg/mL (recalculated on dry leaf powder) was incubated for 24 h. Control cells were grown without the lipid mix and extract. Then, the cell medium was discarded, and the cells were rinsed with PBS. Following this, the cells were scraped out from the cell-growing flasks and centrifuged in PBS buffer at 300× *g* for 5 min. Then, the supernatant was discarded, and the cells were resuspended in a MiR05 buffer at a concentration of 5 million cells per mL.

### 4.8. High-Resolution Respirometry

#### 4.8.1. Chemicals

The mitochondrial respiration medium MiR05 (pH 7.1 at 37 °C) included 0.5 mM ethylene glycol-bis(2-aminoethyl ether)-N,N,N′,N′-tetraacetic acid (EGTA), 3 mM non-aqueous magnesium chloride (MgCl2), 60 mM K-lactobionate, 20 mM taurine, 10 mM monopotassium phosphate (KH_2_PO4), 20 mM 4-(2-hydroxyethyl)piperazine-1-ethanesulfonic acid (HEPES), 110 mM D-sucrose, and 1 g/L of bovine serum albumin (BSA). All chemicals for MiR05 were obtained from Sigma-Aldrich. For the detection of HepG2 cell mitochondrial respiration, the reagents and inhibitors used for the modified SUIT-036 protocol [63] included malic acid, palmitoyl carnitine, cytochrome C, pyruvic acid, succinic acid, adenosine diphosphate, glutamic acid, carbonyl cyanide 3-chlorophenylhydrazone (CCCP), rotenone, and antimycin A. Except for adenosine diphosphate, obtained from Merck (Darmstadt, Germany), all chemicals were purchased from Sigma-Aldrich (St. Louis, MO, USA).

#### 4.8.2. HepG2 Cell Permeabilization

The determination of the amount of digitonin needed for HepG2 cell permeabilization was based on a previously established protocol for digitonin titration aimed to induce maximal cell membrane permeabilization without disruption of mitochondrial respiration [54]. In brief, 1 mL of the HepG2 cell suspension in MiR05 buffer (5 million cells per mL) was injected into an Oxygraph-2k chamber (O2k, Oroboros Instruments, Innsbruck, Austria). Then, 2 µL of 0.2 mM rotenone, 20 µL of 10 mM succinate, and 10 µL of 2.5 mM adenosine diphosphate (ADP) were added, and the stable oxygen flux signal was monitored for 10 min after each chemical addition. When the substrates provided a stable oxygen flux signal, digitonin (10 mg/mL) was added in 2 µL titration steps (up to 4 steps). After each step, the oxygen flux was recorded for 2–5 min, and the action was repeated until the signal reached a maximum and further injections of digitonin did not increase the respiration rate. This experiment determined the amount of digitonin as 8 µL for 1 mL of cell suspension.

#### 4.8.3. High-Resolution Respirometry Measurements of Oxygen Consumption in the FAO Pathway with Permeabilized HepG2 Cells

Studies on the fatty acid oxidation (FAO) pathway were conducted using high-resolution respirometry with two double-chambered Oxygraph-2k machines (O2k, Oroboros Instruments, Innsbruck, Austria), following a modified SUIT-036 protocol [63] (Bioblast, MitoPedia, https://www.bioblast.at/index.php/SUIT-036_O2_mt_D089, accessed on 5 September 2024). The protocol included the addition of H_2_O before cytochrome C. Measurement of mitochondrial respiration was conducted at 37 °C, and after adding the sample, 8 µL of digitonin at a concentration of 10 mg/mL was added, after which the chemicals were added in the following order: adenosine (5 mM) to stimulate the oxidation of residual endogenous substrates; malate (1 mM) for the production of sustained oxidation of palmitoylcarnitine; palmitoylcarnitine (10 µM), for the measurement of fatty acid oxidation-dependent oxidative phosphorylation capacity (F(N) pathway) and (F-OXPHOS); H_2_O (2 µL); cytochrome C (10 µM), to test the integrity of the mitochondrial outer membrane; malate (0.2, 0.5, 1.0, and 2.0 mM titration) and pyruvate (5 mM), to support NADH-linked respiration; glutamate (10 mM), as an additional substrate for the FN pathway (FN-OXPHOS); succinate (10 mM), to reconstitute convergent respiration through the FNS pathway (FNS-OXPHOS); uncoupler carbonyl cyanide m-chlorophenyl hydrazine (CCCP) in 0.5–1 μM steps, for the determination of the electron transfer capacity (FNS-ET); rotenone (0.5 µM), as a CI inhibitor; and antimycin A (2.5 µM), as a CIII inhibitor. A stable oxygen flux signal was ensured before each step to allow for accurate data recording. The oxygen concentration (µM) and oxygen flux (pmol/s × mL) were recorded in real time using DatLab software (Oroboros Instruments). The ET pathway capacities were calculated by subtracting the O_2_ flux before substrate addition from the O_2_ flux after substrate addition [54] as follows: F-OXPHOS capacity was determined using low concentrations of malate from the FAO substrate (palmitoylcarnitine); FN-OXPHOS used palmitoylcarnitine from glutamate; FNS-OXPHOS used palmitoylcarnitine from succinate; and FNS-ET used succinate from the uncoupler (CCCP).

### 4.9. Statistical Data Analysis

Data are presented as the mean ± standard deviation (SD). Statistical significance was set at *p* ≤ 0.05. Data were assessed for normal distribution. A one-way ANOVA followed by post hoc Tukey’s or Dunnett’s tests was used for data comparison. All statistical analyses and graphical presentations were performed using GraphPad Prism (Version 7.0; GraphPad Software, Inc., San Diego, CA, USA).

## 5. Conclusions

Our investigations affirm the positive antioxidant and hepatoprotective effects of nettle leaf water extracts, and we recommend the future development of dry extract or liquid formulations with precisely quantified chemical compositions.

In our study, nettle extracts increased FN-OXPHOS activity and mitigated the fatty acid-induced decrease in FN-OXYPHOS activity. Our data are the first to demonstrate the significance of mitochondria-related respiration effects in the mechanism of nettles’ hepatoprotective properties. Thus, we provide novel data on the impact of nettle leaf water extracts on hepatic steatosis via an improvement in oxygen consumption in the FAO pathway at mitochondria complex I.

## Figures and Tables

**Table 1:** Polyphenol content in the nettle leaf extracts.

Sample	PA	BA	CoA	FA	ChA	CA	R	SA
Average ± SD (μg/mL)								
Es	1.07 ± 0.18	0.14 ± 0.02	0.18 ± 0.01	1.29 ± 0.02	30.9 ± 3.24	10.32 ± 1.14	0.40 ± 0.01	0.85 ± 0.03
Em	0.51 ± 0.05	0.09 ± 0.02	0.13 ± 0.07	1.25± 0.07	37.04 ± 3.9	10.19 ± 0.13	0.66 ± 0.01	0.78 ± 0.03
LOD (mg/L)	0.17	0.17	0.04	0.48	0.68	0.07	0.12	0.11
LOQ (mg/L)	0.51	0.50	0.13	1.44	2.05	0.2	0.35	0.34

Abbreviations: Es, extract obtained via sonication; Em, extract obtained via maceration; PA, protocatechuic acid; BA, 4-OH-benzoic acid; CoA, o-coumaric acid; FA, ferulic acid; ChA, chlorogenic acid; CA, caffeic acid; R, rutin; SA, sinapic acid. The limits of detection (LOD) and quantification (LOQ) are defined as the lowest concentration of the analyte that can be reliably detected and quantified with a 95% probability of obtaining a correct result.

**Table 2:** Vitamin contents in the nettle leaf extracts.

Sample	Riboflavin	Pantothenic Acid	Ascorbic Acid	Pyridoxine	Nicotinic Acid	Nicotinamide
Average ± SD (μg/mL)						
Es	0.49 ± 0.02	0.21 ± 0.01	4.77 ± 0.37	0.039 ± 0.003	0.090 ± 0.011	0.16 ± 0.01
Em	≥0.03	0.23 ± 0.02	4.13 ± 0.51	0.064 ± 0.006	0.103 ± 0.006	0.14 ± 0.03
LOD (μg/mL)	0.03	0.03	0.16	0.008	0.014	0.02
LOQ (μg/mL)	0.09	0.09	0.19	0.023	0.044	0.06

Abbreviations: Es, extract obtained via sonication; Em, extract obtained via maceration. The limits of detection (LOD) and quantification (LOQ).

**Table 3:** Mineral mass concentrations in the nettle extracts.

Sample	Li	Mn	Fe	Ni	Cu	Zn	Rb	Sr	Ba	W
Average ± SD (μg/L)										
Es	9 ± 1	1309 ± 12	3726 ± 12	36 ± 1	93 ± 1	709 ± 23	30 ± 3	1348 ± 18	297 ± 3	1628 ± 23
Em	8 ± 1	1227 ± 26	3530 ± 3	26 ± 1	99 ± 1	677 ± 53	41 ± 4	1402 ± 10	319 ± 5	1213 ± 50

Abbreviations: Es, extract obtained via sonication; Em, extract obtained via maceration.

## Data Availability

The original contributions generated for this study are included in this article; further inquiries can be directed to the corresponding author.

## Supplementary Materials

The following supporting information can be downloaded at https://www.mdpi.com/article/10.3390/plants14070992/s1, Figure S1. UV chromatogram (G1) of nettle extract analysis obtained via sonication; Figure S2. UV chromatogram (G2) of nettle extract analysis obtained via maceration; Figure S3. UPLC-PDA chromatogram profile (I3) at 320 nm in nettle extract obtained via sonication; Figure S4. UPLC-PDA chromatogram profile (I4) at 320 nm in nettle extract obtained via maceration.
