# Supplementary material for: Nettle Leaf Water Extracts for Hepatoprotection: Insights into Bioactivity and Mitochondrial Function
Source: Plants (Basel). 2025 Mar 21;14(7):992. doi: 10.3390/plants14070992 (PMC11990370; doi:10.3390/plants14070992)

Supplement

Figure S1. UV chromatogram (G1) of nettle extract analysis obtained via sonication. Ion chromatograms include peaks indicating content of riboflavin (A1), pantothenic acid (B1), pyridoxine (C1), nicotinamide (D1), nicotinic acid (E1), and ascorbic acid (F1).

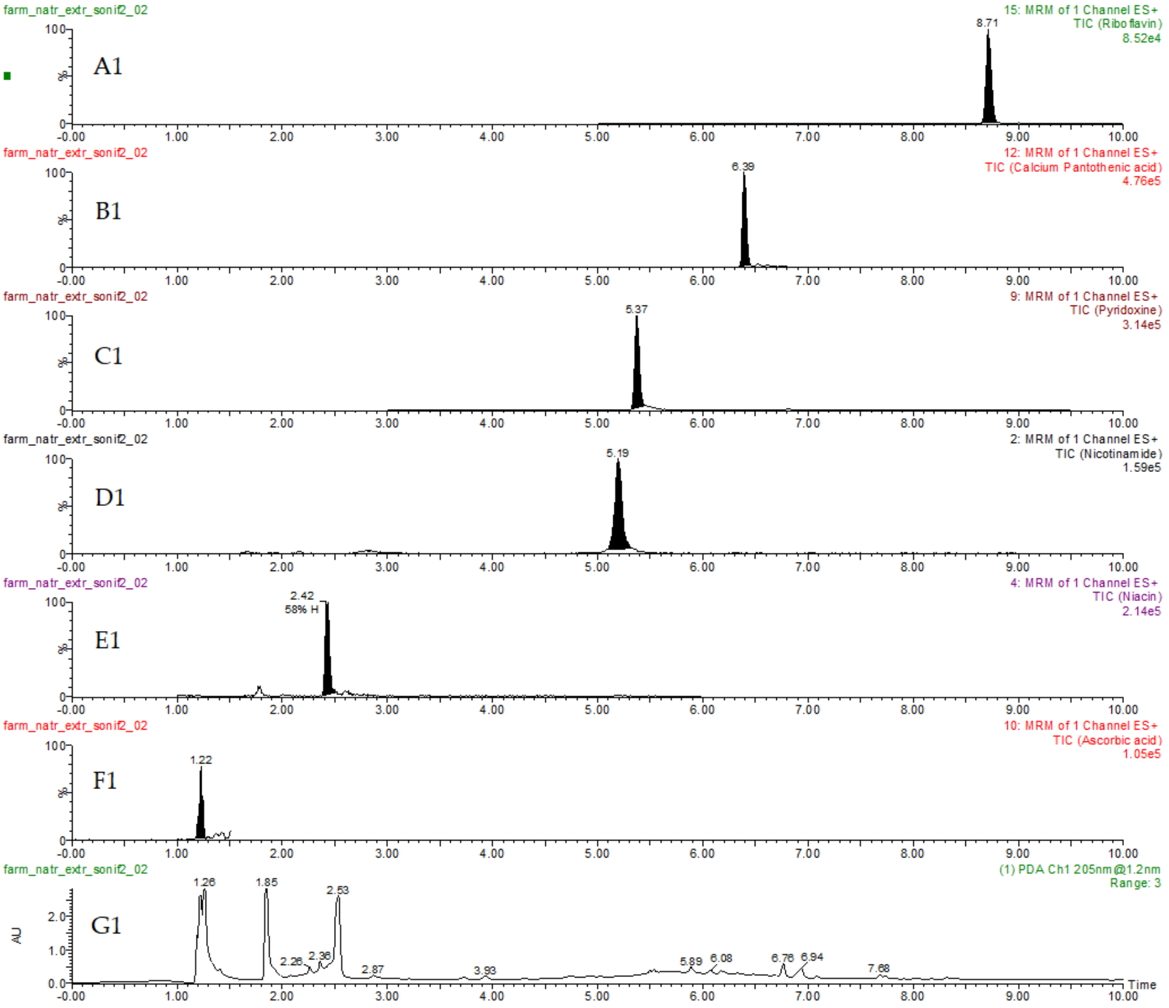

Figure S2. UV chromatogram (G2) of nettle extract analysis obtained via maceration. Ion chromatograms include peaks indicating content of riboflavin (A2), pantothenic acid (B2), pyridoxine (C2), nicotinamide (D2), nicotinic acid (E2), and ascorbic acid (F2).

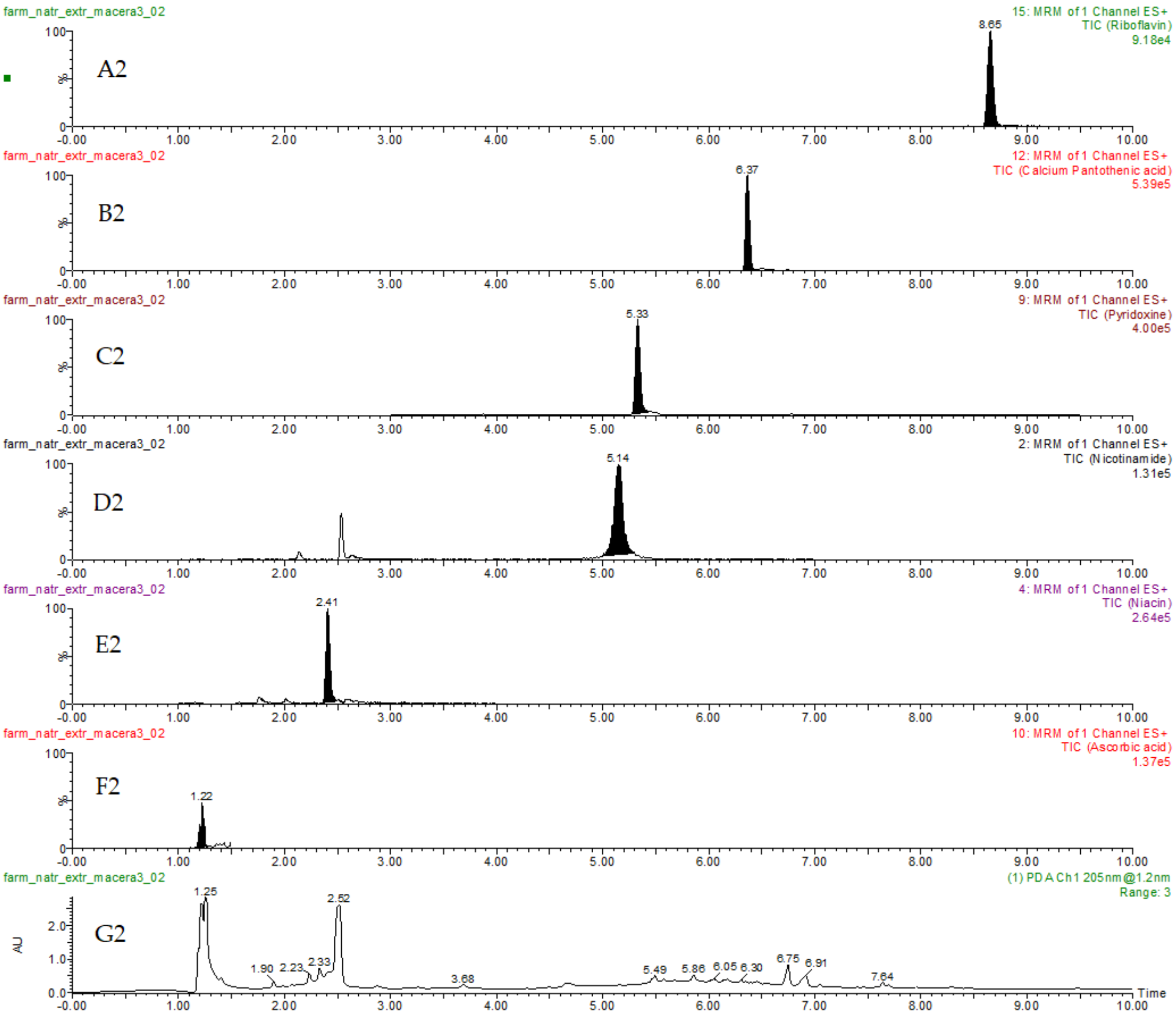

Figure S3. UPLC-PDA chromatogram profile (I3) at 320 nm in nettle extract obtained via sonication. Ion chromatograms include peaks indicating content of rutin (A3), sinapic acid (B3), ferulic acid (C3), o-coumaric acid (D3), caffeic acid (E3), chlorogenic acid (F3), 4-hydroxybenzoic acid (G3), and protocatechuic acid (H3).

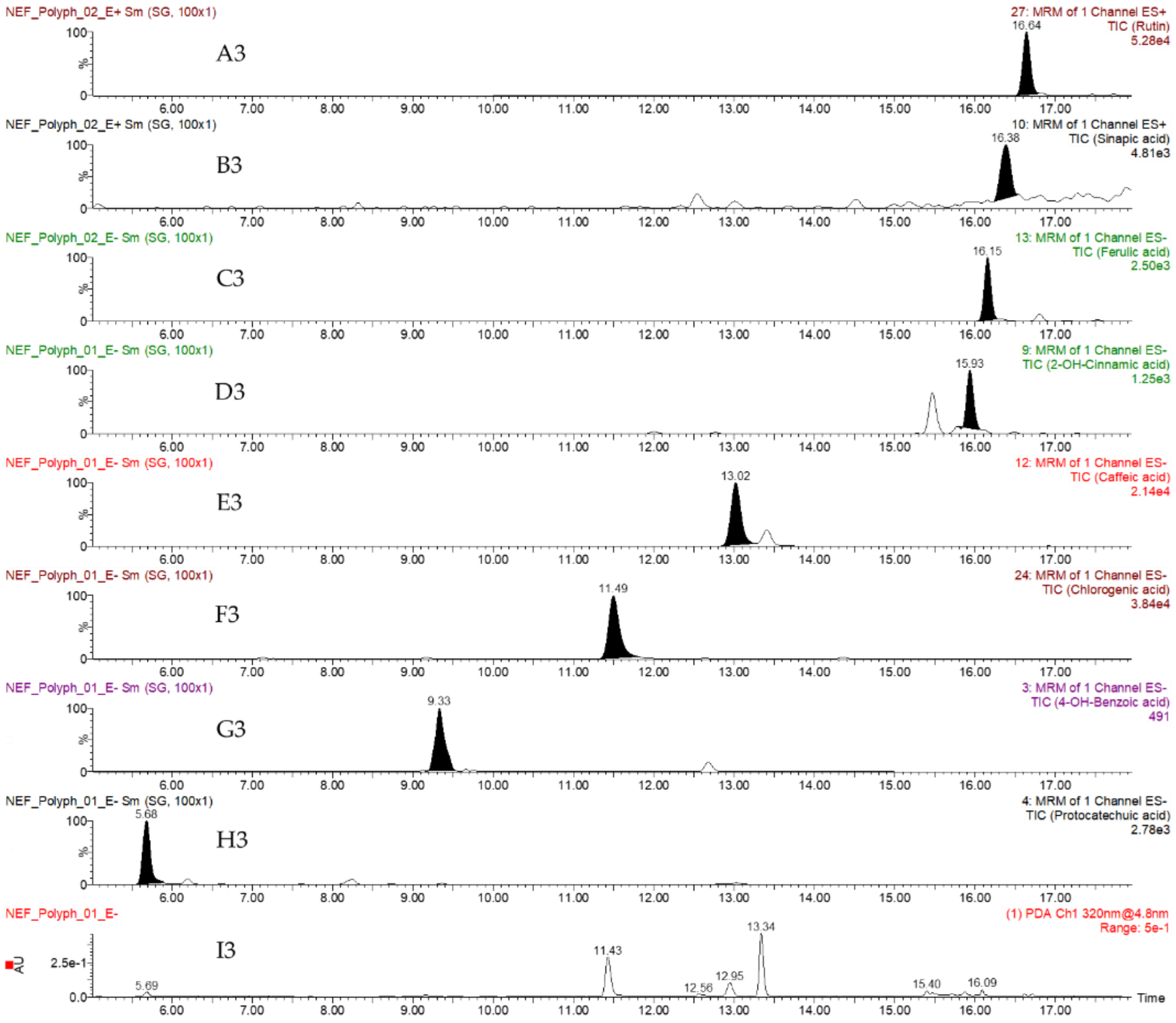

Figure S4. UPLC-PDA chromatogram profile (I4) at 320 nm in nettle extract obtained via maceration. Ion chromatograms include peaks indicating content of rutin (A4), sinapic acid (B4), ferulic acid (C4), o-coumaric acid (D4), caffeic acid (E4), chlorogenic acid (F4), 4-hydroxybenzoic acid (G4), and protocatechuic acid (H4).

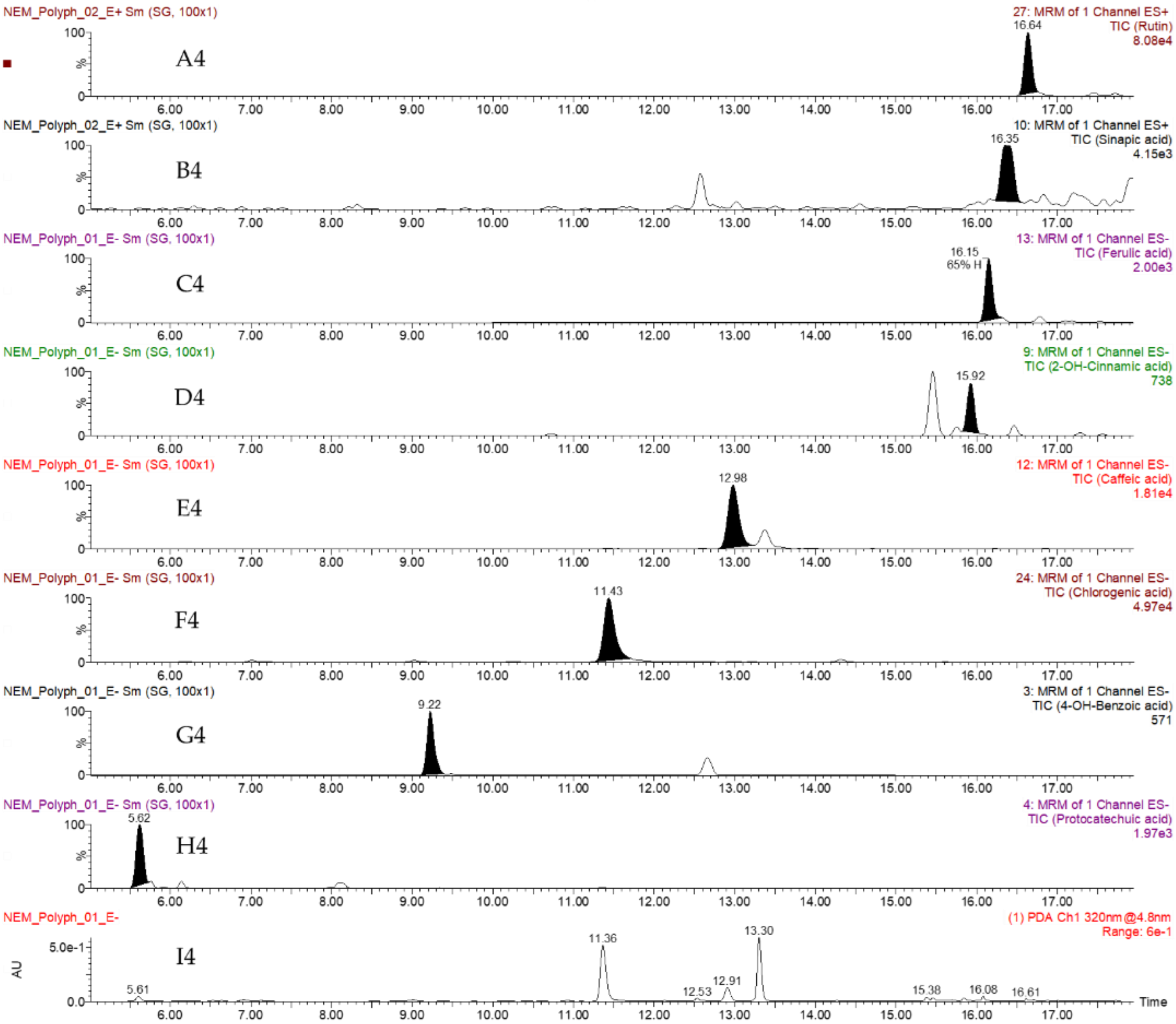

Supplement: Supplementary file 1 [file plants-14-00992-s001.zip › plants-3502693-supplementary.pdf]
